# Potential Application of Electronic Olfaction Systems in Feedstuffs Analysis and Animal Nutrition

**DOI:** 10.3390/s131114611

**Published:** 2013-10-29

**Authors:** Anna Campagnoli, Vittorio Dell'Orto

**Affiliations:** Department of Health, Animal Science and Food Safety, Università degli Studi di Milano, Via Celoria 10, Milan 20133, Italy; E-Mail: vittorio.dellorto@unimi.it

**Keywords:** feedstuff analysis, animal nutrition, feedstuff volatile compounds, electronic odour sensing systems

## Abstract

Electronic Olfaction Systems (EOSs) based on a variety of gas-sensing technologies have been developed to simulate in a simplified manner animal olfactory sensing systems. EOSs have been successfully applied to many applications and fields, including food technology and agriculture. Less information is available for EOS applications in the feed technology and animal nutrition sectors. Volatile Organic Compounds (VOCs), which are derived from both forages and concentrate ingredients of farm animal rations, are considered and described in this review as olfactory markers for feedstock quality and safety evaluation. EOS applications to detect VOCs from feedstuffs (as analytical matrices) are described, and some future scenarios are hypothesised. Furthermore, some EOS applications in animal feeding behaviour and organoleptic feed assessment are also described.

## 1. Introduction

Feed analysis is one of the most important topics in animal nutrition research. Once the nutritional requirements of an animal have been established, a diet that provides the correct balance of nutrients can be formulated if accurate information on feedstuffs is available. Thus, a primary interest of nutritional analysts has been the development of methods designed to quantify the nutritional value of feedstuffs and furthermore, prevent commercial fraud [1]. During the last twenty years, the term “nutritional evaluation”, as applied to feed analysis, has assumed a new, more complex meaning. In addition to the assessment of vitamins, minerals, proteins, lipids, and other essential nutrients, the digestibility, bioavailability, functional properties, palatability, safety, and traceability of feed are also emphasised, with a focus on a more complete interpretation that may be referred to as a “total quality evaluation”. Consequently, the characterisation of specific chemical and physical entities as well as contaminants and undesirable compounds in the feed are of greater scientific and commercial importance. Furthermore, the need for global feed supply traceability, the high-throughput testing demands of the feed industry, and regulatory enforcement have driven an increased need for feed analysis and, consequently, extremely high volumes of required analyses. To meet this demand, simplified rapid analytical methods that are non-destructive and cost-effective for use in high-volume routine analytical assays are needed [2].

As analytes, Volatile Organic Compounds (VOCs) can be considered for these purposes. VOCs comprise a very large group of molecules that can be defined as low-boiling-point and high-vapour-pressure substances that are present in the gaseous state at standard temperature and pressure. VOCs are produced by plants, microorganisms, animals, and some anthropogenic activities. Numerous VOCs are classified as toxic substances; thus, a number of countries have enacted regulations to limit the atmospheric concentrations of some VOCs. However, most innocuous scents or odours are also VOCs; thus, these compounds are utilised as analytes in a variety of research and industrial applications, such as assessing the quality, safety, and organoleptic properties of food and feed [3]. There are two approaches for analysing VOCs. Known VOCs can be individually recognised and/or quantified (e.g., by gas chromatography or gas chromatography-mass spectrometry). Gaseous analyte mixtures can be identified by a unique aroma signature that can be defined as a “fingerprint” [3,4].

The need to more precisely quantify and express the aroma characteristics of VOCs that have been released as mixtures from specific sources has necessitated the development of methods and instruments that are capable of recording unique quantitative and qualitative measurements of headspace volatile compounds derived from samples of interest. Electronic Olfaction Systems (EOSs) appear to be interesting candidates for this purpose [3]. Many characteristics that directly determine the effective quality and/or safety of a feed are often aspects of or described by its aroma. Thus, the previously mentioned “total quality evaluation” of feedstuffs, which requires the simultaneous recognition, classification, and/or quantification of several parameters, could be at least partially achieved via a method based on the features and properties exhibited by EOSs. Consequently, feedstuffs analysis and animal nutrition represent fields in which EOSs could be applied for both research & development purposes and practical in-field applications (e.g., in farm and feed industry contexts and in production plants).

For a more precise definition of EOSs, it is first necessary to define an Electronic Nose (EN) as an instrument and as an analytical approach. Gardner and Bartlett [5] defined an EN as an instrument that comprises an array of electronic chemical sensors with partial specificity and an appropriate pattern-recognition system that is capable of recognising simple or complex odours. This definition could imply that electronic noses are comprised of parts that include arrays of non-specific solid-state gas sensors, a variety of transducers, data collectors, and data analysis tools, all of which are oriented to the classification (and, under some circumstances, quantification) of chemical clusters of volatile compounds, including, in particular, odours [6]. The term “Electronic Nose” is also used to name a group of systems that differ from that described above, such as mass spectrum-based systems, ion mobility spectrometers, electron capture detectors, and quadruple fingerprint mass spectrometers [4,7]. Some authors maintain that the use of EN to define the latter analytical techniques is incorrect [4,8]. These techniques are not to be considered EN in the strictest sense because they not provide a collective data output from a sensor array and were originally designed to detect and identify individual components of a gas mixture [4]. However, some recent applications of these techniques have been oriented towards the evaluation of volatile mixes to characterise their overall aroma rather than identify/quantify a single analyte. Thus, other authors use the terms “Sensor Array Technology” [9], “Electronic Olfactometry” [10], “Active Odour Sensing System” [11], and “Artificial Olfaction System” [12], among other synonyms, to refer to a gas sensor system, regardless of the underlying technology [8].

Thus, for the purposes of this article, the term “Electronic Olfaction Systems” will be used to identify electronic instruments that generically mimic, in a simplified manner, animal olfaction functions and that are constituted by not otherwise specified, single or multiple “detection devices” that are able to non-selectively interact with mixtures of odorous molecules to produce signals that are then sent via a recording device to recognition software, which analyses the data and enables pattern recognition. The use of the term “Electronic Nose” will be restricted to the group of EOSs that are specifically equipped with multiple electronic sensor arrays, such as detection devices that include organic polymers, metal oxides (e.g., metal oxide semiconductors), quartz crystal microbalances, surface acoustic waves, catalytic field-effects, and conducting polymer technology [3,4].

EOSs are frequently described as “biologically inspired” because, at least in theory, they are constituted by a structure mimicking the anatomy and physiology of the animal sense of smell based on (a) the activation of peripheral chemosensory receptors by odorant detection (detection device/sensor array activation); (b) transfer of a sensorial stimulus to the brain (transduction and recording of a signal from the detection device to data analysis software); (c) message transformation during olfactory perception and conscious recognition (data extraction, treatment, and interpretation) [13,14].

During the design and realisation of such a complex device, problems of a different nature arise. The realisation of appropriate detection devices/sensors involves issues of a technological nature, and further difficulties arise at the level of electrochemical message encoding and transduction. There are also theoretical problems related to signal treatment and processing and the final classification and interpretation of odours. Thus, although the final device should be able to replicate functions of a biological nature, in the practical realisation of this aim, designers are forced to create a “black-box” type of model, that is, a system that replicates animal functions without having an internal structure that replicates biological anatomy. This occurs both at the level of the reception signal, because it is impossible to replicate the enzymatic mechanisms involved (which are insufficiently understood), and at the level of higher processing centres (in particular, the largely unknown cerebral cortex activities). Only at the intermediate processing level (e.g., at the level of the cerebral bulb) is it possible to mimic the natural structure because this appears to be known with sufficient completeness [13,14].

Furthermore, the complex evolution of the chemical senses in animals has resulted in a sensory apparatus with high smell and taste acuity, which ensures self-nourishment. Such peripheral chemosensory systems functions contribute to determine the perception of nutritional value of feedstuffs. Food ingestion simultaneously evokes odour, taste, and thermo-mechanical (somatosensing) sensations. Consequently, olfaction represents the capacity to identify feed volatile compounds that are predominantly derived from essential nutrients in plants. In contrast to electronic systems, the sensory apparatus for high smell and taste acuity in mammals and avians (farm animals included) is sufficient to contribute, in close relationship with the other chemical senses and other physiological factors, to regulate palatability, feed appetence, feeding behaviours, and feed intake [15]. It is not likely that such a complex system could be completely replaced by an instrument.

For these reasons, the comparison of an electronic odour sensing systems with an animal nose is at best similar to a comparison of the eye of a bee with that of a mammal [16]. Although this is a strong analogy that was proposed some time ago, it is still valid in many cases [7]. Nevertheless, just as a bee eye is blind towards part of the visible spectrum but sensitive to other wavelengths, in the same way, the evaluation of non-odorant volatile compounds can be usefully achieved by EOSs. For this reason, in well-defined cases, the correlation between animal odour impressions and EOS data is reasonable [7]. Thus, this family of devices demonstrates their usefulness in several types of applications. EOSs are more sensitive (have much lower odour detection thresholds) to most VOCs than animals; EOSs generally offer a greater potential for discriminating the individual gases present; and EOSs provide rapid results and operate continuously [3]. All of these features can enable the collection of information that is not otherwise available in many application fields and demonstrates their utility in feedstuffs analyses and in animal feeding behaviour research (which is likely the most complex target EOSs application in animal nutrition).

The aim of this review is to describe the significance (from feedstuff technology and animal nutrition perspectives) of the various VOCs released by the ingredients that are most frequently used in farm animal diets. Furthermore, studies performed with the aid of EOSs in feedstuffs analyses, in quality and safety evaluation, in farm animal feeding behaviour, and in feed organoleptic assessment studies are described.

## 2. VOCs from Forages and EOS Applications

### 2.1. VOCs from Forages and the Application of EOSs in Silage Quality Evaluation

In European countries, North America, New Zealand, and Australia, hay, grasses, whole-crop silage maize, and other cereals and legumes are the major crops used as fresh or conserved forage for the nutrition of ruminants. Silage making is one of the most important sources of conserved forages. In particular, silage forms a basic component of ruminant diets [17]. This approach is widely used for storing forage for feeding milk- and meat-producing ruminants [2]. Silage making has increased considerably since the 1960s and has become economically relevant for many farming systems in temperate areas of the world [18].

Maize (*Zea mays L.*) is the principal ensiled crop. The entire plant is harvested and is considered high-energy forage [19]. Apart from maize, one of the commonest crops usually conserved by ensilage is grass. Both single species of grass (e.g., Italian ryegrass (*Lolium multiflorum L.*) or perennial ryegrass (*Lolium perenne L.*)) and mixed species of grasses and legumes are grown as silage crops [20].

The advantage of forage ensiling is that it makes crops available for feeding throughout the year or during periods of restricted seasonal availability of pastures for grazing animals [21]. Silage production is based on a multi-step natural fermentation, the last phase of which is characterised by anaerobic conditions under which lactic acid-producing bacteria convert water-soluble carbohydrates into organic acids, mainly lactic acid [22].

The preservative effect against the growth of detrimental microorganisms results from a first aerobic phase with acetic acid production and consequent anaerobiosis and acidification (by means of lactic acid synthesis) of the ensiled mass. The growth of lactic acid bacteria is encouraged by creating an anaerobic microclimate by compressing the feed material and can be facilitated by adding starter cultures and/or organic acids [20,23].

The nature and characteristics of the ensiled crops and the environmental conditions and practices adopted during silage making have a strong impact on the resulting nutritional value and fermentation stability [20]. During acidification and conservation, potential oxygen ingress into the silage can cause dry matter (DM) and nutritional losses as well as increase the risk of proliferation of potentially pathogenic or otherwise undesirable microorganisms [24]. Furthermore, from a perspective of nutrient preservation and quality maintenance until feeding, the changes that occur during the aerobic feed-out phase (during which the mature silage is removed from the mass under aerobic conditions and fed to the animals) are equally as important as those that occur during the anaerobic storage phase [21]. Furthermore, the activity of aerobic spoilage organisms may lead to changes in the composition of volatile compounds, thereby affecting fermentation quality.

All changes that occur during the conservation and feed-out phases affect the potential development and final concentration of VOCs other than lactic and acetic acids, which are strongly related to silage quality; these compounds affect the final silage nutritional value, hygienic status, and organoleptic characteristics [25]. Therefore, fermentation quality can be evaluated mainly on the basis of the concentrations of (a) lactic acid (lactic acid is the strongest of all silage organic acids and creates a low pH environment as the result of homofermentative reactions); (b) short-chain volatile fatty acids (acetic, propionic, and butyric acids, which provide aerobic stability during the early phases of the ensiling process (after the closure of the silo bunker) but are also indicators of poor silage quality if present in excess in mature silages); (c) products resulting from the degradation of proteins and amino acids (with ammonia nitrogen as an indicator); (d) fermentation-derived products of water-soluble carbohydrates, such as organic acids, carbon dioxide, and alcohols (e.g., ethanol is an indicator of silage quality because it is produced by anaerobic heterofermentative bacteria that are active during the early phases of the ensiling process and is also an indicator of yeast activity) [26–28]. Furthermore, the buffering capacity in silage is due to the presence of weak organic acids (e.g., citrate, malate, and quinate), orthophosphates, sulphates, nitrates, chlorides, and non-protein nitrogenous compounds in the material, some of which are also detectable as VOCs [20].

This description indicates that several and often interrelated factors may influence silage quality. Thus, adequate assessment of forage quality is an important but complex challenge. The determination of silage quality is normally conducted at the moment of silo opening and is complicated by the fact that it may change due to aerobic deterioration. When oxygen penetrates into the silage during feed-out or storage, acid-tolerant (facultative) aerobic microorganisms, mainly yeasts, moulds, aerobic bacteria, and (under certain circumstances) pathogenic *Clostridia spp.* (which are characterised by a strictly fermentative metabolism) begin to proliferate. These organisms induce substrate oxidation (e.g., of residual sugars, lactic acid, acetic acid, and ethanol) and the synthesis of undesirable VOCs (and numerous other non-volatile molecules) that are associated with instability of the silage environment and that clearly result from oxygen penetration into the ensiled mass. All silages eventually deteriorate in the presence of ox0ygen; however, there is considerable variation in the length of their stability upon exposure to air [29]. Aerobic deterioration reduces the nutritional value of silage due to the degradation of fermentation acids and cell-wall carbohydrates and by the catabolism of protein to ammonia-nitrogen [30]. Thus, aerobic stability is a major component of the general assessment of silage quality. Although the fermentation quality of fresh silages (in closed or recently opened silo bunkers) can be determined with the aid of reference values that assist in the interpretation of the results of chemical analyses, it becomes progressively difficult to objectively determine silage quality and usability once the silo has been opened and spoilage processes have begun.

The consequences of aerobic deterioration include potentially marked changes in the composition of VOCs. Thus, the dry matter intake (DMI) of deteriorated material is often restricted, and animals may refuse the feed completely [20]. For these reasons, in addition to measuring the nutritional constituents and fibre fractions, which are commonly determined for use in feeding management, silages can be evaluated for VOCs that result from fermentation reactions to assess fermentation quality based on the content of undesired degradation products (among the organic acids cited above, acetic and butyric acid are considered the principal markers) [31] and VOCs resulting from the metabolism of undesirable microorganisms (bacteria, fungi, and yeast). However, this evaluation of silage quality during the feed-out phase requires the use of many samples, expensive labour and equipment, qualified personnel, and, most importantly, time-consuming laboratory analyses. Because the results are not directly available, they are often incompatible with the timing of farm activities and thus cannot be used for the daily control of the silage working face. The practical consequence is usually a fast and cost-effective assessment of forage, which is performed by sensory evaluation, *i.e.*, based on smell, colour, temperature, structure, and DM content as directly determined by the human senses [32]. However, this method is subjective and requires skilled personnel. Aerobic stability can be determined under laboratory conditions by continuously measuring the temperature of silages exposed to air for several days at constant ambient temperature [33–35]; however, the unwieldy nature of this approach limits its application. These limitations underlie the difficulty of implementing objective control points for silage management under practical conditions. The demand for effective silage quality and safety assessment has been intensified by the implementation of Commission Regulation (EC) No. 183/2005 “Regulation of the European Parliament and of the council laying down requirements for feed hygiene” [36]. Forages intended as feedstuff for animals that are used for milk or meat production are included because the regulation prescribes compliance with hygiene regulations at all stages of the production of foods of animal origin. Furthermore, European low and scientists emphasise the need for objective control instruments for silage management that can directly provide reliable results.

One possibility to ensure an objective and rapid assessment of forage quality could be the application of EOSs. These systems might represent a valid method of screening of ensiled products both during the conservation stage and during the feed-out phase to differentiate among silage qualities and stage of deterioration on the basis of volatile markers for spoilage processes and to avoid the entry of undesirable substances and organisms such as mould, mould metabolites, and yeast into the food chain [37].

An EN was applied to silage analysis by Masoero *et al.* [38]. Researchers compared the use of near-infrared spectroscopy (NIR) and an EN including 10 metal oxide semiconductors (MOSs) to evaluate dry matter, pH, buffering capacity, and total nitrogen, soluble nitrogen, ammonia, alcohols, lactic acid, and volatile fatty acid content in fresh farm silages. As expected, NIR provided better results than the EN for predicting pH, whereas the EN was superior at estimating total fatty acid and ammonia levels and buffering capacity. Furthermore, the EN exhibited advantages over some other analytical methods, including NIR, for the evaluation of fermentation characteristics. In fact, due to some pre-processing steps, such as drying and grinding, a percentage of VOCs (e.g., organic acids and alcohols) are lost in samples submitted to “classical” routine analyses. Only methods that employ undried samples are sufficiently efficient for these analytes. Furthermore, routine silage analyses using VOCs as fermentation quality markers are considered quite expensive and are usually time-consuming and incompatible with farming needs, particularly with respect to the speed of mass front removal. Masoero *et al.* [38] used an EN as a simple alternative method for evaluating gaseous components. The portability of EN, combined with its lack of a requirement for sample pre-treatment, speed, and low operating cost, represents substantial in-field advantages. In fact, due to these features, despite the lower sensitivity of this approach compared with classical, more diffused, instrumental methods, EN allows the measurement of several samples, which may decrease the total final measurement error due to the reduced variance in sampling. Despite the wide variability of silage samples, EN techniques are of interest for their ability to rapidly, easily, and inexpensively provide information relevant to the stocking and parameter monitoring of farm silages. On the other hand, the different crops, silaging techniques (e.g., silos, nylon bales, barns), and other practices adopted during silage making need the development of specific analytical protocols (in particular, the set-up of sampling techniques and training samples during dataset construction).

### 2.2. VOCs as Forage Odours and the Application of EOSs to Organoleptic Assessment

Huhtanen *et al.* [25] studied the effects of VOCs as markers of variation in silage fermentation quality in the voluntary feed intake of cattle. As confirmed by some authors, such as Muck [39], the main components responsible for the characteristic smell of silages are volatile fatty acids that evaporate quite easily when introduced to air; by contrast, lactic acid has a bland odour and little volatility upon exposure to air. Acetic acid provides silages with their characteristic vinegar odour and taste; propionic acid produces a sharp, sweet smell and taste; and butyric acid produces a rancid butter smell and taste. Elevated levels of butyric acid indicate silage deterioration from secondary fermentation, which in the presence of unpalatable nitrogenous end products, such as amines and amides, may lead to a significant decrease in the DMI and energy level of the forage. Butyric acid and nitrogenous proteolysis are the result of clostridial activity in the silo.

Apart from organic acids, other VOCs should be mentioned. Ethanol is obviously associated with an alcohol smell. Esters often also have characteristic smells. Because esters are known to be odorants, they could affect the taste of silage and, consequently, feed intake. Some authors consider esters more important than organic acids in defining the odour of ensiled mass. Mo *et al.* and Kristensen *et al.* [40,41] expected esters to contribute to silage flavour due to their volatility. Furthermore, many esters have low odour thresholds and thus are perceived at concentrations of parts per million. Ethyl lactate, which is characterised by a creamy odour with hints of fruit, has a weak negative influence on DMI. Esters can be the most abundant class of VOCs in red clover silages [42] and in grass silages [40], with ethyl esters being the predominant subclass of all esters [42]. Some authors have observed that ethyl acetate and ethyl lactate show a strong correlation with ethanol in fresh and well-fermented silages. Further studies on other molecules of this chemical class, as methyl esters might be required [43].

Some yeast species produce various end products that are responsible for objectionable off-odours. Yeast can produce alcohol mixed with acetic acid, which generates a vinegar odour, a stinging smell that sometimes causes cows to refuse silages; other yeast end products include methyl and ethyl acetates, which resemble the smell of “fingernail polish remover”. Combinations of these gaseous substances can also induce feed refusal [44].

As described conspicuous amounts of various VOCs are synthesised inside ensiled masses. Table 1 lists the principal components of silage smell.

Mo *et al.* [40] identified more than 50 different fermentation products in grass silages. Attempts have been made to identify a relationship between silage quality and intake for unspoilt silages [45,46]. Because the composition of VOCs may change in a few days as a result of aerobic spoilage caused by oxygen ingress, it is difficult to attribute changes in DMI to a single fermentation product.

Consequently, an ideal analytical method for screening should be capable not only of analysing each molecule but of identifying and classifying the characteristic patterns generated by multiple volatile compounds. EOSs could theoretically answer this problem. An EN was tested with the aim of both confirming a correlation between the DMI and volatile composition of silage and verifying the ability of the EN to evaluate silage quality. Roß *et al.* [47] conducted an experiment with maize and grass silages samples by analysing the samples using methods commonly used for microbiological and chemical (comprising aerobic spoilage) assessment. Furthermore, the samples were evaluated using quartz microbalance sensor technology with and without thermal desorption pre-treatment (to increase measurement sensitivity). Fermentation qualities, hygiene status, stage of deterioration, and preference behaviour by goats and DMI of silages with different lengths of aerobic exposure were compared, based on the signal pattern obtained from the EN. Chemical and microbial composition were correlated with the signals given by the chemosensor system and with the feed assumption. In particular, the authors observed (a) a relationship between changes in the sensor pattern and changes in the composition of the silage gas and (b) a direct relationship between the sensor signals and the concentration of the measured gases. Consequently, when samples were divided into two qualitative classes, “fresh silage” (0 days of air influence) and “deteriorated silage” (8 days of air exposure), the EN was able to correctly classify all samples. For silages in good condition, the sensors mainly responded to aromatic components, whereas in deteriorated silage, the sensors exhibiting the highest response were mainly sensitive to alcohol compounds.

A further topic of interest is the attempt to define hay and grass flavour and its relationship with animal preferences. Starting from ryegrass (*Lolium multiflorum*) hay as a matrix, Aii *et al.* [48] confirmed experimentally by gas chromatography and mass spectrometry the traditional knowledge that the flavour of hay obtained from sun-cured material is more intense than heat-dried material of the same matrices. Drying treatments markedly affected the flavour constituents. A greater concentration of decomposition products was generated by heat drying. Furthermore, storage duration affected the aroma, generally by reducing the odour intensity. After a long storage period, hays obtained using different drying methods (sun-curing or heat drying) are similarly perceived by animals.

Akakabe [49] described the characteristics of Italian ryegrass hay. Italian ryegrass hay and silage share a characteristic aroma. Aldehydes and alcohols appear to contribute to hay odour, which has been defined as “green leaf like” [48,49]. The role of other compounds in the final characteristic odour of hay was also noted. Some esters and lactones, one anhydride, and one norterpene contribute importantly. In fresh silage comprising the same vegetal variety, organic acids are the most important contributor to the characteristic sweet-sour aroma. Furthermore, some of these organic acids are also responsible for off-odours in stale silage. Accordingly, when Italian ryegrass hay and silage were compared in a feeding trial performed with cattle, hay was preferred, followed by fresh silage and stale silage, in that order. Finally, it can be hypothesised that cattle exhibit a preference for a high amount of aldehydes and alcohols; high amounts of low-molecular-weight organic acids, which mainly develop in stale silage, appear to explain the poor palatability of this last forage.

## 3. VOCs from Concentrates and EOS Applications

Interest in EOSs began to increase in the late 1990s, when the technology had developed sufficiently to enable practical applications. Moreover, during the same period, significant attention was given to methods for the early detection of changes in food quality and in animal feed quality [50–52]. In particular, grain safety became a topic of interest. Among the causes of grain damage and loss in quality, the negative effects of mould were, and remain, among the most investigated. Consequently, the application of EOSs has been explored extensively [53].

Substantial grain damage is manifested as general spoilage, nutritional loss, the formation of mycotoxins and potentially allergenic spores [54]. During the 1990s, the most commonly used screening method to detect fungal growth and other objectionable odours in grains in international and most national trades was human sensory analysis [51,55]. Alternatively, toxigenic fungi and the related mycotoxins contamination of cereal grains can be detected and quantified using complex extraction procedures and analytical techniques [56,57]. Thus, alternative screening methods that were inexpensive, simpler, more efficient, and preferably as rapid as human sensory analysis but less subjective and hazardous for human operators were needed; this need drove the first attempts to employ an EN to investigate grain safety and the causes of grain spoilage [51,52,58–60].

Moulds that cause spoilage produce a complex range of volatile compounds, which are mixed with other volatile compounds that are not necessarily considered markers of grain quality. Even in the 1970s, Kaminski *et al.* [61,62], demonstrated that fungi that cause spoilage produce volatile compounds that are characteristic and different from those produced by bacteria or the grains themselves. Advantageously for the detection of undesirable substances, starch and cellulose in grains accumulate volatile metabolites, act as natural adsorbents, and enable the tracking of past fungal growth in cereal-based feed and food [52]. Many authors have noted the potential application of volatile compounds in the classification of grains based on background odours produced by different cereals. Among the many compounds with different chemical characteristics (hydrocarbons, alcohols, ketones, aldehydes, esters, *etc.*) that influence cereal odour [63], fungal sesquiterpenes are particularly useful for indicating mycotoxin formation [64,65]. Moreover, sesquiterpenes appear to be unique for each fungal species [52,53]. However, some authors have reported differences in volatile compounds produced by secondary metabolism in similar strains depending on whether they are produced from *in vitro* cultures or from naturally contaminated grains; this finding suggests that substrates can influence the synthesis of volatile compounds [52,53].

Furthermore, fungi commonly produce VOCs as they begin colonising nutrient-rich substrates such as grain; however, from a biological perspective, the reasons to produce volatile compounds can be numerous. Consequently, different biological pathways with different related products can be involved. The production of VOCs might be a way of removing inhibitory intermediates from metabolism under unfavourable conditions. Volatile compounds might also have inhibitory effects on other fungi and act as self-regulators of growth and development [53]. Thus, the metabolic pathways leading to the formation of VOCs in naturally contaminated grain could provide important insights into the relationship between various groups of volatile compounds and mycotoxins, although elucidating these pathways remains challenging. In fact, one of the major difficulties in research on this topic concerns the identification of consistent VOCs as markers for in-field applications.

Despite the complexity of the proposal, research began in the 1990s and in the first half of the last decade by attempting to verify the ability of an EN to detect volatile compounds as an indicator of the potential for grain spoilage. The main aims were efficiently summed up by Magan and Evans [53] as follows: (a) to verify the range of fungal volatile compounds produced by spoilage fungi; (b) to identify the presence of volatile compounds in naturally contaminated grain as an early indicator of spoilage; (c) to investigate the possible relationship between odour discriminators and volatile compounds produced by spoilage fungi on seed; (d) to approach the early detection of spoilage mould activity on grain substrates; and (e) to verify the potential for using EN technology to detect seed spoilage. These aspects have been investigated by numerous authors [53,66–68].

Thereafter, the results of these studies enabled researchers to focus their interest on more complex targets, such as the detection of mycotoxigenic fungi or mycotoxin contamination [57,58,65,69–73].

The most recent experiments have focused on a semi-quantitative/quantitative evaluation of mycotoxin concentrations in contaminated grains. Cheli *et al.* and Campagnoli *et al.* [74,75] conducted pilot experiments to evaluate the potential of a 10-MOS EN for the rapid identification of maize samples contaminated by aflatoxins. Using linear discriminant analysis with a cross-validation procedure as the classification model, the instrument classified all 30 samples correctly into two groups, contaminated (from 7 to 100 μg·kg^−1^ total mycotoxin concentration) and non-contaminated (<4 μg·kg^−1^ total mycotoxin concentration). Furthermore, Eifer *et al.* and Campagnoli *et al.*, both in 2011 [76,77], verified the application of two different ENs (based on quartz microbalances and MOS, respectively) as simple and rapid methods to detect and differentiate *Fusarium* species and deoxynivalenol (DON), respectively as contaminants in wheat grain. The results obtained by Campagnoli *et al.* allowed the authors to classify the analysed samples into three classes on the basis of the European Union limits for DON in unprocessed durum wheat: (a) non-contaminated; (b) contaminated below the limit (DON < 1,750 μg/kg); and (c) contaminated above the limit (DON > 1,750 μg/kg). A further comprehensive study was conducted by Gobbi *et al.* [54]. This study used a 6-MOS chemical sensor array EN to study maize cultures inoculated with different species of fumonisins-producing *Fusarium* fungi. The instrument was able to classify the inoculated maize culture samples according to their fumonisin content. Unlike other cited experiments, in which multivariate classification models were applied, Gobbi *et al.* used a regression model (partial least squares) in their quantitative approach.

As general consideration, the identification of mycotoxins and their quantification appears to require the careful selection and extraction of features from the signals produced by the EN sensors to achieve the desired results. For instance, Campagnoli *et al.* [77] observed that a quite complex approach to EN signal selection based on Principal Component Analysis was necessary. Only by starting from this premise and providing a sufficient amount of data can the next step required to solve such complex problems be achieved, *i.e.*, the choice of an adequate data analysis algorithm (e.g., classification or regression multivariate models) or more powerful artificial intelligence algorithms (*i.e.*, Artificial Neural Nets, Support Vector Machines, *etc.*).

Furthermore, it might be possible to use EOSs or EN technology for the early detection of insect odours in grains. However, these odours are less easily replicated than fungal ones [53]. Indeed, there are fewer studies on this topic than those dedicated to mould and fungal metabolites. Volatile compounds associated with insect damage are different from those associated with normal grain by human testers [53]. Only one experiment has described the specific use of an EN (MOS-based) to recognise cereal insect contamination. The experiment analysed wheat samples categorised by five different levels of insect damage. Each group was recognised and discriminated from the others using linear discriminant analysis [78].

The applications of EOSs to the investigation of the potential causes of cereal damage are listed in Table 2.

The identification of undesirable materials in feed ingredients represents another application of EOSs. For instance, the detection and classification of processed animal protein (PAP), which has been banned as an ingredient for farm animal feedstuffs in the EU and in several other countries, is one such application.

By exploiting differences in the odours of animal and vegetable ingredients, Campagnoli *et al.* [79], verified the potential for a 10-MOS sensor EN for PAP detection and recognition in feed. Reference feedstuffs were used for the experiment. A compound feed for bovines was fortified with different types of PAP (meat and bone meal, fish meal, and both). Three different levels of inclusion of PAP were considered (0%; 0.5%, and 5%). The EN was able to recognise samples containing a very low level of PAP, distinguishing between samples containing 0.5% PAP and blank samples. The level of discrimination reached a sufficiently low level of inclusion that it can be considered for the screening of raw materials (also in the feed industry), although two limitations of the experiment were apparent. First, only one type of compound feed was adopted as a matrix for the dispersion of the undesirable substance, thereby reducing potentially negative effects on the discrimination power of the method. Furthermore, it was not possible to differentiate samples containing the higher level (5%) of fish meal when jointly present with meat and bone meal from samples containing fish meal exclusively, possibly due to the masking of the meat and bone odour by the fish meal odour. A similar topic was investigated by Hui *et al.* [80], who confirmed the ability of a unique sensor for nitrogen compounds using an electronic system to predict fishmeal freshness.

As demonstrated previously in Section 2, for forages, the aromatic characteristics of concentrates can affect feed intake in farm animals. Despite the important role of odour and other organoleptic characteristics in palatability, the literature is lacking with respect to DMI and the quality of animal-derived products and the characterisation and role of VOCs present in concentrates used as ingredients for farm animal rations. The most complete list of information related to VOCs in concentrates and their effect on DMI is found in Rapisarda *et al.* [81,82]. The authors focused on 15 different ingredients (beet pulp; oat grains; dehydrated alfalfa; soybean hulls; soybean meal 44 and soybean meal 49; sunflower meal; barley meal; corn gluten meal; wheat bran; corn middlings; canola meal; wheat grains; corn grains; and pea grains). For each of these ingredients, a preference test was conducted using adult ewes and lambs. A very interesting definition of the VOCs for each ingredient was achieved using a double approach. First, gas chromatography-olfactometry and gas chromatography-mass spectrometry were used to recognise volatile compounds. The later part of the experiment was performed using a mass spectrometry-based EOS, which was adopted to assess the “global” characteristic aroma profile of each ingredient to verify the effect of feed odour on animal preferences and to confirm the ability of the EOS based analytical methods to correctly classify ingredients based on their palatability.

A total of 217 different molecules from 10 different chemical classes were recognised. Aldehydes were characterised by an orange, green hay, or garlic odour; esters were associated with a fruity smell; various lactones were associated with a garlic or peach aroma; pyrazines were associated with a burnt or nutty smell; sulphur compounds were associated with a garlic, meat, mushroom, or “cooked potato” aroma; and heterocyclic compounds were associated with smells ranging from sweet to spicy. As expected, terpenes, as ketones, were characterised by a wide range of different intense flavours ranging from floral to sweet, coconut or fruity sensations, whereas amines exhibited their notorious rotten fish odour. Some alcohols were also detected.

Signals from the EOS, which generated a volatile fingerprint for each feed, defined the effect of each ingredient due to its characteristic combination of VOCs. The number of VOCs in each chemical class and the level of animal preference for feedstuffs emitting these compounds are reported in Table 3.

The EOS differentiated fairly well between palatable and unpalatable feeds for lambs and ewes in the preference test. Feedstuffs classified as unpleasant by the EOS and refused in the animal preference test were characterised by the presence of sulphur compounds and methanamine. Conversely, aldehydes and terpenes (with the exception of pinene, which contained a negative resin-pine note) characterised the samples classified as pleasant and having green and fruity flavour notes.

Furthermore, EOS enabled some complementary conclusions related to animal preferences that would be arduous to obtain using other techniques. For instance, a principal component analysis based on EOS data showed greater similarity between feeds preferred by lambs than between discarded feeds. The authors concluded that barely weaned lambs most likely enjoyed fewer odours and only some specific volatile compounds that are held in common by pleasant ingredients. The EOS results demonstrated that adult ewes enjoy a greater variety of odours than lambs. Furthermore, a principal component analysis based on EOS data enabled the simultaneous recognition and differentiation of feed preference level in association with their nutritive properties. Thus, high-protein feeds were differentiated from starchy feeds and from feeds high in neutral detergent fibre and low in starch. This last observation could lead one to assume some ability in animals to recognise the nutritive properties of food on the basis of feed odour itself; however, further data are necessary to confirm this hypothesis. Using a single method, it was possible to screen data of different types (the principal nutritional features for each feed in association with their characteristic flavours) that otherwise would require a multi-analytical panel.

## 4. Conclusions and Perspectives

As demonstrated by the extensive bibliography regarding food analyses and control, there is widespread interest in the present and future applications of EOSs. However, despite numerous studies of the application of EOSs to food and the similarities between the fields of feed technology and animal nutrition, fewer applications of EOSs have concerned the control of feedstuffs and animal nutrition.

The results of the application of EOSs to grain science, which is a facet of food sector research, are actually relevant for both food and feed analysis. Thus, the limited number of bibliographical data and the limited results described in feedstuffs analysis and animal nutrition could also be due, at least in part, to the poor implementation of research in these specific sectors. Wilson [3] has written an interesting review on the applications of ENs in the agriculture and forestry sectors, further demonstrating the success of techniques based on artificial olfaction in fields other than food technology that are nonetheless related to feed technology and animal nutrition.

Further explanation for why EOSs are not more widely used in the farm animal sciences might be related to the initial cost of instruments, which might be judged too high for a sector in which high-throughput analyses are considered essential. Furthermore, analytical methods must be robust to be applied in the field. Many studies describing EOSs relevant to the topics of interest of this review were described by their authors as “pilot” or “preliminary” studies because they were based on sample datasets that were not very large compared with the number of variables under study (e.g., the number of sensors constituting an EN array). The same set of data is often applied during both the training phase and as the validation set using cross-validation procedures. Certainly, the use of an adequate number of samples that describe the real complexity of the problem of interest and strict validation techniques could increase the robustness of the method.

Moreover, from a technical perspective, it is worth emphasising that a number of failures to obtain satisfactory results at the research level and in practical applications in many fields might be due to the lack of awareness of the importance of device selection in achieving successful results.

Given the technical limitations of EOSs, we could define them as they are, “an attempt to mimic the principles of smelling that gives another view on the whole scene of volatiles compared to its biological inspiration” [7]. Thus, from a practical perspective, detection device/sensors transducer selection must be performed on a case-by-case basis; then, the appropriateness of the entire EOS for the particular application must be evaluated. Some key considerations involved in the selection of EOSs for a particular application must necessarily include assessments of the selectivity and sensitivity range of individual detection device arrays for particular target analyte gases (which are likely present in the samples to be analysed), the number of unnecessary (redundant) sensors with similar sensitivities, as well as sensor accuracy, reproducibility (preciseness), response speed, recovery rate, robustness, and overall performance [3,7].

A fundamental design concept for an effective array of sensors is that each sensor should maximise overall instrument sensitivity and provide different selectivity profiles over the range of target gas analytes to be detected or classified for a particular application [14,83]. Ideally, for an EN, a sensor array should consist of individual sensors that produce different responses to a given odour analyte such that a unique aroma pattern is created. If it is difficult to obtain unique aroma patterns for different gas analytes, the sensor selection must be modified or the number of sensors must be adjusted when classification, performance, cost, or technological limitations are issues of concern [3].

Performance can also be improved by adding additional transducers based on different chemical and physical principles to the sensor array, such as gravimetric, thermal, and optical sensors, which have completely different transduction principles. Additional sensors with new sensitive layers are also under development, e.g., sensors based on DNA, molecularly imprinted molecules, or immobilised natural receptors [7,15]. This means that current technologies and their future improvements will permit the development of more effective tools and techniques that are targeted and satisfactory in their application, *i.e.*, more “fit to purpose”. In addition, EOSs generally have the potential to be more portable than complex analytical laboratory instruments because they do not require chemical reagents, provide rapid results, permit non-destructive analysis, and are inexpensive for a single analysis [4,77,84].

Thus, EOSs have far greater potential for use by unskilled personnel for practical in-field applications (e.g., in the farm and feed industries, where affordability and ease of use are crucial) [4].

The potential applicability of EOSs as a screening tool that can reduce the number of samples needed for subsequent, more complex, and expensive confirmatory analyses has been demonstrated [4,77]. Furthermore, EOSs may be used to verify samples requiring a qualitative assessment in which the final sample assessment is based on a discrimination criterion, e.g., “acceptance” or “rejection”. This approach has already been adopted for certain food or feed batches of raw materials [4,77,85]. The more interesting examples in this article could be considered the testing and/or classification of the quality (and safety) of silages and grains. As emphasised in Section 2, the numerous silage VOCs appear to be adequate analytes for use in EOS applications; furthermore, EOSs represent an adequate analytical approach for field conditions. The ability to differentiate among silage qualities using an electronic device fulfils a requirement of silage management and could theoretically enable the implementation of critical control points. The final aims are as follows: reducing the risk of losses in quality due to malfermentation or aerobic deterioration, improving forage safety, and contributing to the protection of animal health and productivity.

Finally, for some farm species, feed odour is sometimes more important than taste. As abundantly demonstrated, the smell of food cannot be simply measured through the determination and quantification of individual VOCs but is represented by patterns of many volatile compounds that characterise a specific and typically complex overall effect; therefore, the smell of food must be analysed using techniques that are specifically designed for this purpose [84,85]. Thus, EOSs might be useful in feed preference studies. However, the sensitivity characteristics of sensor transducers and biological receptors remain far from comparable. In particular, the complexity of the superior cerebral skills and abilities of animals and humans remain inimitable. In other words, it is not possible to capture the “significance” of a flavour fingerprint or “to recognise” odours using an electronic device [86]. Thus, with current technologies, efforts to arrive at a universal device that can finely discriminate between flavours, perfumes, and smells and eventually replace the animal nose have been disappointing [87]. For these reasons, the application of EOSs in feed preference studies should not be considered a substitute for the animal sense of smell but rather as a complementary approach that is able to reveal information that is otherwise not appreciable. For example, when a sensor transducer array is profitably chosen, the data generated by electronic devices can enable the extraction of features that have been “cleaned” of redundancy. Practically, this advantage can be applied to the analysis of complex mixes of different odour substances, potentially enhancing the ability to recognise a single ingredient in a mixture of feeds or to enhance the ability to differentiate between different levels of quality of the same feed. In other words, the “measurement accuracy” attainable by EOSs could help to overcome misinterpretations in feed preference tests that result from imprecision of the animal senses (e.g., a specific feed odour recognition failure or the lack of recognition of one odour when mixed with others). Furthermore, EOSs could enable the detection of non-odorant molecules [3] that are implicated in animal self-nourishment and that are not otherwise observable [88,89]. The results obtained using this approach could be combined with those obtained from other analytical methods and behavioural studies and applied to develop a better understanding of animal feeding habits and preferences.

## Figures and Tables

**Table 1.** Characteristic smells recognised by humans and the principal chemical classes detected in silages.

**Chemical classes**	**Molecules**	**Odour profiles**
Volatile fatty acids	Acetic acid	Vinegar
	Propionic acid	Sharp sweet
	Butyric acid	Rancid butter
	Lactic acid	Odourless or slight; not unpleasant
Ammonia nitrogen and nitrogenous end products	Ammonia nitrogen	Ammonia odour
	Amines	Putrid, fishy, ammonia-like
	Amides	Rum
Alcohols	Ethanol	Pleasant, alcoholic
Esters	Methyl acetate	Nail polish remover
Ethyl acetate		Agreeable odour, rather sweet and like “pear drops”
Ethyl lactate		Creamy odour with hints of fruit

**Table 2.** The applications of EOSs to investigations of the causes of cereal damage.

**Main Topic**	**Application Area**	**References**
Detection of volatile compounds as indicators of potential grain spoilage	- Detection methods for mycotoxins in the food chain	[50]
	- Fungal volatile compounds as indicators of food and feed spoilage	[51]
	- Potential use of EN for the detection of volatile compounds as indicators of fungal activity and to differentiate between species	[52]
	- Detection methods for moulds in food spoilage	[54]
	- Moulds presenting off-odorous compounds on oatmeal	[55]
	- Potential application of EN to the assessment of cereal quality	[60]
	- Detection of contaminants in bulk grain using sensors and physical methods	[71]

Detection of mycotoxigenic fungi in contaminated grains	- Evaluation of wheat contamination by *Fusarium poae* fungi based on EN response	[56]
	- Detection of ochratoxin and deoxynivalenol in barley grain by gas chromatography–mass spectrometry and EN	[57]
	- Evaluation of mycotoxins in food using biomolecular/electronic techniques	[58]
	- EN detection of fungal volatile compounds from trichodiene in naturally infected wheat and triticale grain	[65]
	- Mycotoxins, ergosterol, and odorous volatile compounds in durum wheat during granary storage	[69]
	- Artificial olfactory system for the discrimination of grain quality	[70]
	- Detection and differentiation between mycotoxigenic and non-mycotoxigenic strains of *Fusarium* spp. using volatile profiles	[73]

Semi-quantitative/quantitative evaluation of mycotoxin concentrations in contaminated grains	- Use of an EN for the prediction of high and low fumonisin contaminations in maize	[53]
	- Use of an EN for the classification of aflatoxins in maize on the basis of their concentration	[74]
	- Detection and classification of aflatoxins in maize using an EN	[75]
	- Differential detection of potentially hazardous *Fusarium* species in wheat grains using an EN	[76]
	- Use of an EN for the recognition and classification of durum wheat naturally contaminated by deoxynivalenol	[77]

Early detection of insect odours in grains	- Detection of age and insect damage in wheat using an EN	[78]

**Table 3.** Animal preferences for feed ingredients and the number of molecules detected for each chemical class.

	**Animal Preference**		**Number of Molecules Recognised for Each Chemical Class**									

**Feed**	**lambs**	**ewes**	**Al**	**Am**	**Ket**	**Es**	**Lac**	**Pyr**	**Sul**	**Ter**	**Heter. comp**	**Total Number of Recognised Compounds**
Soybean meal 49	1st	12th	4	0	2	0	1	2	5	0	0	14
Wheat grains	2nd	3rd	5	0	1	0	0	0	0	0	0	6
Pea grains	3rd	2nd	2	0	1	0	0	0	1	1	0	5
Corn grains	4th	4th	3	0	1	0	0	0	0	1	0	5
Soybean hulls	5th	10th	8	0	4	0	1	1	2	3	0	19
Beet pulps	6th	1st	11	0	4	0	2	1	2	5	2	27
Wheat bran	7th	6th	4	0	2	0	0	1	5	1	0	13
Soybean meal 44	8th	9th	6	1	4	0	1	1	4	1	0	18
Corn middlings	9th	7th	5	0	2	0	0	0	5	0	0	12
Canola meal	10th	13th	2	0	0	0	2	0	3	0	0	7
Sunflower meal	11th	11th	5	1	3	1	0	0	5	1	0	16
Corn gluten meal	12th	5th	7	0	2	0	0	0	4	1	0	14
Dehydrated alfalfa	13th	14th	6	0	3	1	1	1	5	3	0	20
Oat grains	14th	8th	10	0	3	1	1	0	2	7	2	26
Barley meal	DNS	DNS	8	0	3	0	2	0	1	0	1	15

Modified from Rapisarda *et al.*, [82]. Animal preferences (expressed as the level of DMI, mg/kg BW, in 6-min tests) are shown using ordinal numbers. 1st is the most preferred; 14th is the least favoured. Chemical class abbreviations: Al.: Aldehydes; Am.: Amines; Ket.: Ketones; Es.: Esters; Lac.: Lactones; Pyr.; Pyrazines; Sul.: Sulphur-containing; Ter.: Terpenes; Heter. Comp.: Heterocyclic Compound; DNS: Data not shown.
